# Implementation of thyroid eye disease registry in Iran: rationale and research protocol

**DOI:** 10.1186/s13023-024-03053-9

**Published:** 2024-02-06

**Authors:** Shadi Akbarian, Abbas Sheikhtaheri, Farid Khorrami, Hossein Ghahvechian, Nasser Karimi, Mohsen Bahmani Kashkouli

**Affiliations:** 1 Skull Base Research Center, Eye Research Center, The Five Senses Institute, Iran University of Medical Science, Tehran, Iran; 2 Department of Health Information Management, School of Health Management and Information Sciences, Iran University of Medical Sciences, Tehran, Iran; 3 Health Information Technology, Faculty of Paramedicine, Hormozgan University of Medical Sciences, Bandar Abbas, Iran; 4 Skull Base Research Center, Eye Research Center, The Five Senses Health Institute, Iran University of Medical Sciences, Rassoul Akram Hospital, Tehran, Tehran 1465544814 Iran

**Keywords:** Thyroid eye disease, Thyroid eye disease registry, Iran, Registries, Study protocol

## Abstract

**Background:**

To describe the implementation of a registry system for patients with thyroid eye disease (TED) in Iran to obtain more information about its nature, prevalence, and annual incidence, as well as extend insight into the etiology, pathogenesis, and eventually make an accurate prognosis of different medical or surgical treatment methods.

**Methods:**

After receiving approval from the Disease Registry Committee of Iran University of Medical Sciences (IUMS) in 2019 and the Ministry of Health and Medical Education (MOHME) in 2020, the protocol was introduced in three consecutive phases at regional, provincial and national levels. The establishment of a registry committee in Rassoul Akram Hospital, one of the medical centers affiliated to IUMS, was the first step to organizing the registry project's main core. The steering committee included six subgroups of required subject fields. The members are experts in developing a guideline, providing a new dataset, drawing an outline for the next steps, and structuring user-friendly software through several panel discussion meetings. The data is collected from clinical and para-clinical/imaging findings, laboratory evaluations, and their selected treatment strategy, retrospectively and prospectively.

**Results:**

The purpose is to broaden our knowledge about the profile of TED; accordingly, data related to patients’ demographics, thyroid gland disease (status, duration, treatments, and function tests), general medical and ocular history, along with visual/ocular exams resulting TED status are collected and recorded in a 2- language software. The web-based software system is accessible at https://orc.iums.ac.ir. To maintain data security, prioritized user access was defined for different members. Furthermore, diverse methods, such as employing trained staff and utilizing software validation rules, were implemented to control data quality in every step of data collection, entry, and registration. Medical records of retrospective subjects were also evaluated and entered after accuracy verification.

**Conclusion:**

Iran's TED registry provides practitioners with comprehensive data on natural history and phenotype variations in clinical features and outcomes. It facilitates patient recruitment and, consequently, earlier diagnosis on a large scale which helps improve treatment and quality of life for patients.

## Background

Thyroid eye disease (TED) is an autoimmune inflammatory disorder with a wide range of ocular signs and symptoms and unforeseen clinical course. [1] It is the most common orbital disease worldwide and the most prevalent cause of axial exophthalmos, which is predominantly reported in females, unlike its more severe indications in males. [2–4] In other words, the female-to-male ratio was found to be 6:1 in affected cases, but in severe forms, this was decreased to 4:1, particularly in men over 50. [5, 6] TED is an autoimmune disorder of orbital soft tissue usually associated with thyroid dysfunction, mainly hyperthyroidism or Graves’ disease. Although it can also manifest in hypothyroidism or the absence of dysthyroidism (Euthyroid status), studies have asserted that orbital retrobulbar tissue is less involved in these patients than the ones in hyperthyroid status. [7]

The incidence and prevalence of TED have been reported in different countries, such as those of Germany [8–10], Wales [11, 12], Spain [13], USA [14], Singapore [15], South Korea [16], Japan [17], Denmark [18], Sweden [19] and Ghana [20]. It has also been investigated in the Iranian population in recent years, and 36% of all thyroid patients have been reported, of which 90% indicated hyperthyroidism in their primary status. [21–25] Since there is insufficient data on the characteristics and natural history of TED in our country, setting up a registry system for such a disease seemed high priority. A patient registry applies observational study methods to compose comprehensive evidence regarding clinical features of all cases of a particular condition or all subjects of a specified disease in a specific population. Not only does a registry system provide information about the prevalence and other epidemiologic characteristics, as well as environmental and genetic factors of a health-related condition, but also it is a practical tool to estimate the prognosis of different applied therapeutic methods to evaluate behavioral patterns of a region’s population and monitor outcomes. It is, indeed, “an organized system for collection, storage, retrieval, analysis and dissemination of information on individual persons who have either a particular disease, a condition (e.g., a risk factor) that predisposes [them] to the occurrence of a health-related event, or prior exposure to substances (or circumstances) known or suspected to cause adverse health effects” according to the definition by “National Committee on Vital and Health Statistics in the United States”. [26–28] Recently, the beneficial application of outcome data in clinical registries has introduced them as a valuable tool for improving the significance and quality of healthcare for affected individuals. Consequently, an ever-increasing number of patient registries has been reported worldwide, particularly for eye diseases. [29] IRIS registry, known as the only ongoing TED registry worldwide, has released a brief report [30] (2013–2018) from a large American national clinical population on TED prevalence and associated factors in 2020 ARVO annual meeting abstract. At present, among 284 running patient and health outcomes registries supervised by medical universities in Iran, three are thyroid-associated programs, and thirteen are proceeding in the field of ophthalmology, according to the latest update of the Iran Ministry of Health and Medical Education’s website. This is the first time to conduct a registry for thyroid eye disease in Iran to reach more detail and facts about these patients, particularly prevalence and annual incidence, as well as extensive insight into the etiology and pathogenesis and eventually make an accurate prognosis of different methods in medical or surgical treatment. The purpose of this report is to describe the protocol for the implementation of this registry in Iran.

## Method and result

### Design and population

In this registry, the main purpose is to determine the natural history of TED disease, including demographic characteristics as well as ethnicities and age/gender groups, provide data on the prevalence and annual incidence of patients at distinct levels of TED involvement, and to facilitate patient recruitment and participation in clinical practice with various geographical/genetic features to explore the best therapeutic planning in collaboration with other national research parties.

First, the protocol was introduced to the Disease Registry Committee of Iran University of Medical Sciences (IUMS) for evaluation in 2019, and then approved by the Disease Registry Committee of Iran Ministry of Health and Medical Education (MOHME) in 2020. To fulfill targets, different consecutive phases were scheduled by the steering committee, including system development/regional (phase 1), provincial (phase 2), and national implementation (phase 3):

*Phase 1* Establishment of a steering committee in Rassoul Akram Hospital, the main referral center for TED patients, to provide a guideline for the TED registry, develop a dataset, develop an efficient software system, and outline the framework of the whole project, as well as recording data of currently and retrospectively referred TED patients to this center.

*Phase 2* Participating hospitals in Tehran will start data collection and registering TED patients while all data is rechecked by two members, one optometrist, and one ophthalmologist, from the steering committee.

*Phase 3* In panel discussion sessions conducted by our national committee, selected ophthalmic research centers and academic hospitals around the country are invited to join the registry team for patient recruitment in other provinces.

### Steering committee and governance

**Fig. 1:**
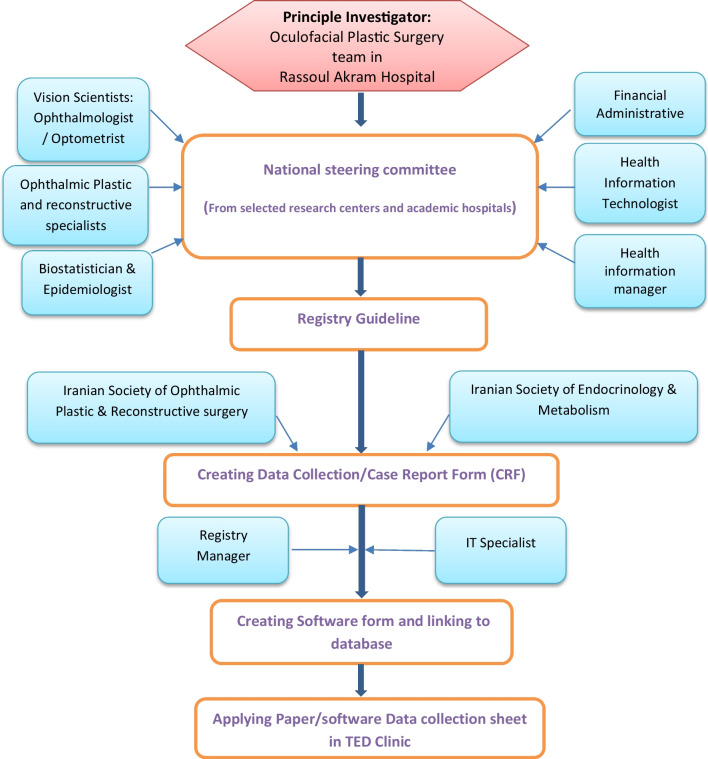
Flow diagram of the process and roles in the Iranian national TED registry

In 2020, the IUMS was authorized by MOHME to be the coordinating center for the TED registry since Rassoul Akram Hospital, affiliated to IUMS, has been recognized as the main referral center for this disease. Hence, stakeholders were identified, and a committee was established by the faculty members from different departments, including ophthalmology, biostatistics and epidemiology, health information management, financial administration, and health information technology (IT). Committee members are recognized as the principal to supervise, evaluate, and develop the registry so several meetings were organized to develop the protocol, predict possible limitations of defined process implementation as well as provide a list of alternative solutions, define and delegate various responsibilities for operational subgroups, and regulate sessions not only to schematize the future of the program but also to assess the progress and quality of accomplished steps. The flowchart in “Fig. 1” displays an overview of TED registry process and roles.

### Patient recruitment and registry implementation

According to the last updated registry guideline, the executive manager and trained staff are in charge of patient recruitment. Patients are not registered unless TED is approved when two of the three following signs are demonstrated [1]:

1. The patient has been recently treated due to previously diagnosed Graves’ or Hashimoto’s disease or in the presence of thyroid autoantibodies in hematological examinations with no thyroid dysfunction.
2. Common ocular symptoms: unilateral or bilateral eyelid retraction with temporal flare (with or without lagophthalmos), unilateral or bilateral proptosis (comparing previous patient’s photographs), restrictive deviations with specific typical patterns, compressive optic neuropathy, intermittent eyelid edema or erythema, chemosis and/or Caruncle edema.
3. Radiographic signs include spindle enlargement of one or more of the inferior rectus, medial rectus, superior rectus and/or whole lifting muscles, and the lateral rectus.

If there are only orbital signs, the patient is monitored for other orbital diseases or the development of thyroid disease in the future.

Enrolled patients undergo clinical examinations by any project partners as board certified ophthalmologists. Additional laboratory or para-clinical tests and imaging are requested if further assessment is necessary for the final diagnosis. Follow-up sessions’ duration is also considered depending on the activity and severity of the disease.

### Inclusion/exclusion criteria

Inclusion Criteria: Approved TED according to the above-mentioned conditions at any age.

**Fig. 2:**
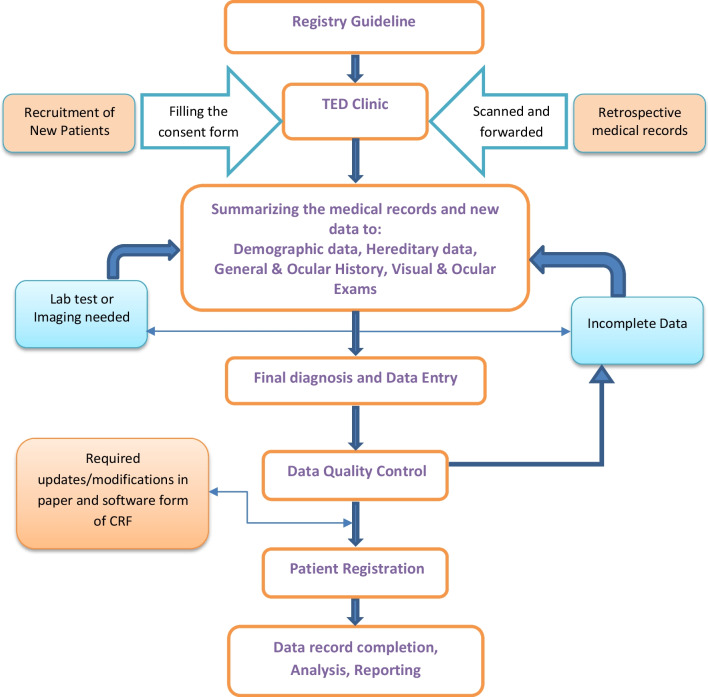
Flow diagram of the patient registration processes in the Iranian national TED registry

Exclusion Criteria: If the patient's signs and symptoms can be attributed to a disease other than TED. The process of patient recruitment to final registration is illustrated in “Fig. 2”.

#### Data set development

**Fig. 3:**
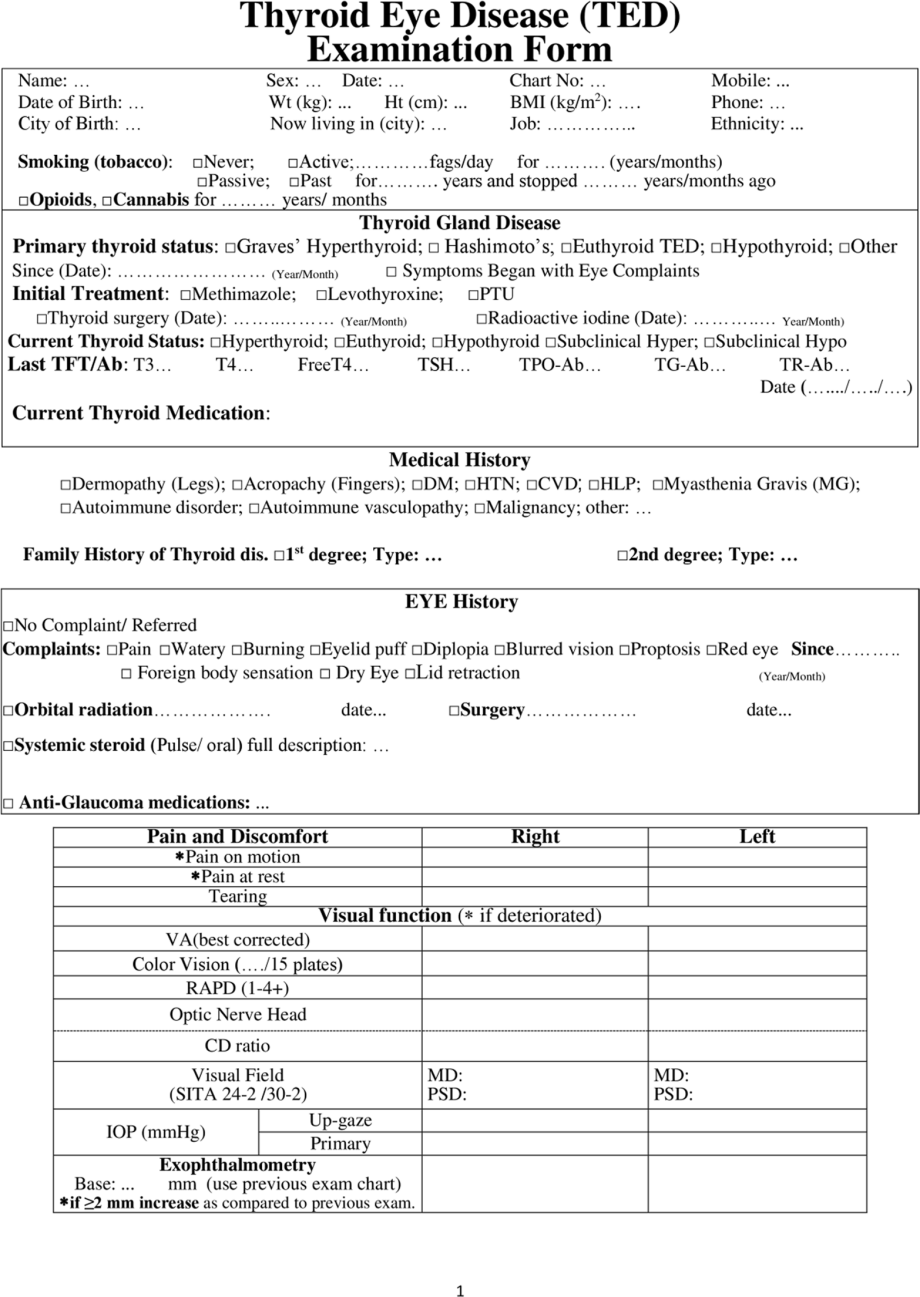

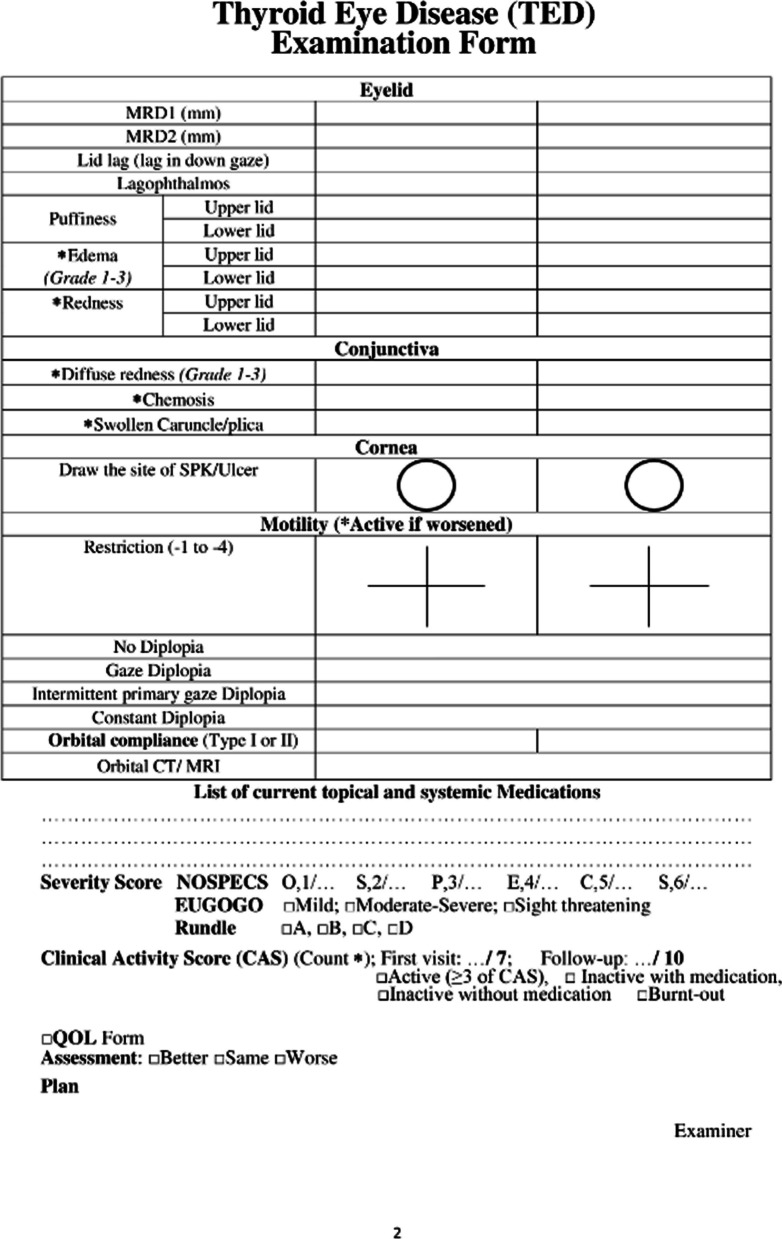
Final version of the TED form introduced for the nationwide application

After developing the steering committee, the TED case report form was suggested. The original TED form has been in use in Rassoul Akram Hospital since 2005. In 2010, the Iranian society of ophthalmic plastic and reconstructive surgeons and the Iranian society of endocrinology and metabolism published a book entitled "Diagnosis and treatment of thyroid eye disease". Since then, the form has been revised and modified annually. We used this form to develop the case report form for the registry. In addition, we considered available case report forms identified through the review of kinds of literature, textbooks, and other related clinical registries. Then, the extracted data were compared to the initial reporting form and discussed in several steering committee meetings. TED form was then nationally introduced for a uniform data entry (Fig. 3).

Eventually, the modified form of TED was finalized, containing 72 variables in six categories of demographic data and smoking status, thyroid disease status, history of other systemic/ocular disease and medications, findings from a comprehensive examination of eye and adnexa, and consequently TED status in terms of activity and severity as well as treatment considerations. These sections and their subcategories are detailed in “Table 1”.

**Table 1:** Sections and the containing variables of case report form

*Categories*	*Data elements*
Demographic data and smoking status	Name*, Last name*, Father’s name, Gender*, File number*, National code*, Weight, Height, Date of birth* (age), City of birth, City of location^¶^, Telephone, Mobile^¶^, Smoking* (Tobacco, Opioids, Cannabis), Smoking* (active, passive, past), Ethnicity
Thyroid gland disease	Primary thyroid status*, Onset of disease*, Initial treatment*, History of Thyroid gland surgery*, History of receiving Radioactive Iodine*, Current thyroid status*, Current thyroid medication*, Last thyroid function/antibodies test (T3, FreeT4, TSH, TPO-Ab, TG-Ab, TR-Ab)
General medical History	Dermopathy*, Acropachy*, Diabetes, Hypertension, Hyperlipidemia, Myasthenia Gravis, Cardiovascular disease, Autoimmune disorders, Autoimmune vasculopathy, Malignancy, Current topical and systemic Medications 1st/2nd degree relatives’ involvement of thyroid disease (yes/no)* and its type*
Ocular history	Eye pain*, Watery eye*, Burning*, Eyelid puff*, Diplopia*, Blurred vision*, Proptosis*, Red eye*, Foreign body sensation*, Onset of disease*, History of Orbital radiation* and any Eye surgery*, Full description of Systemic Steroid consumption*, Anti-Glaucoma medication usage
Visual and ocular exams data	Pain on motion*, Pain at rest*, Tearing*, Best corrected Visual Acuity*, Color vision*, RAPD*^¶^, Optic nerve head* (pink/sharp, pale, edematous), Cup/Disc ratio*, Visual Field (MD^¶^, PSD^¶^), IOP^¶^ in primary gaze*, IOP in up-gaze, Exophthalmometry, MRD^¶^1*, MRD2*, Lid lag* (lag in down gaze), Lagophthalmos*, Puffiness* (upper/lower lid), Edema* (upper/lower lid), Redness* (upper/lower lid), Diffuse redness* (Grade 1–3), Chemosis*, Swollen Caruncle/plica*, Corneal status* (normal, SPK, Ulcer), Motility restrictions in 4 directions*, Diplopia* (Gaze, Intermittent Primary, Constant),Orbital compliance* (i/ii),
TED status	Severity Scores of NOSPECS*/ EUGOGO*/ Rundle*, Clinical Activity Score* and Status*, Assessment and Plan

^*^Starred elements are the mandatory fields to complete

^¶^City of location: currently living city; Mobile: mobile phone number RAPD: relative afferent pupillary defect; MD: mean deviation; PSD: pattern standard deviation; IOP: intra-ocular pressure; MRD: margin to reflex distance

#### Software development and security

**Fig. 4:**
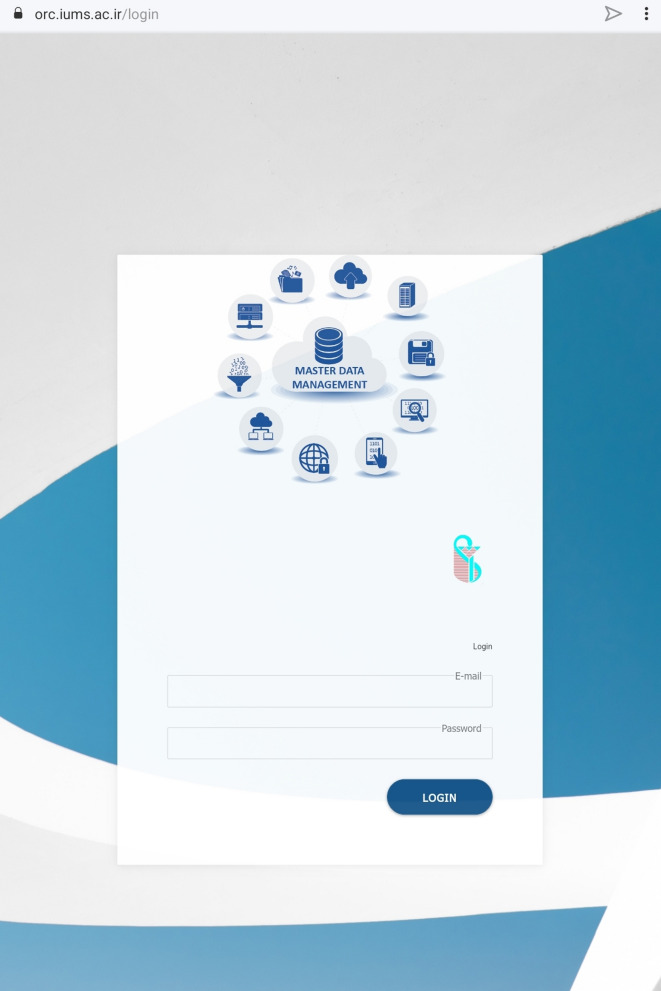

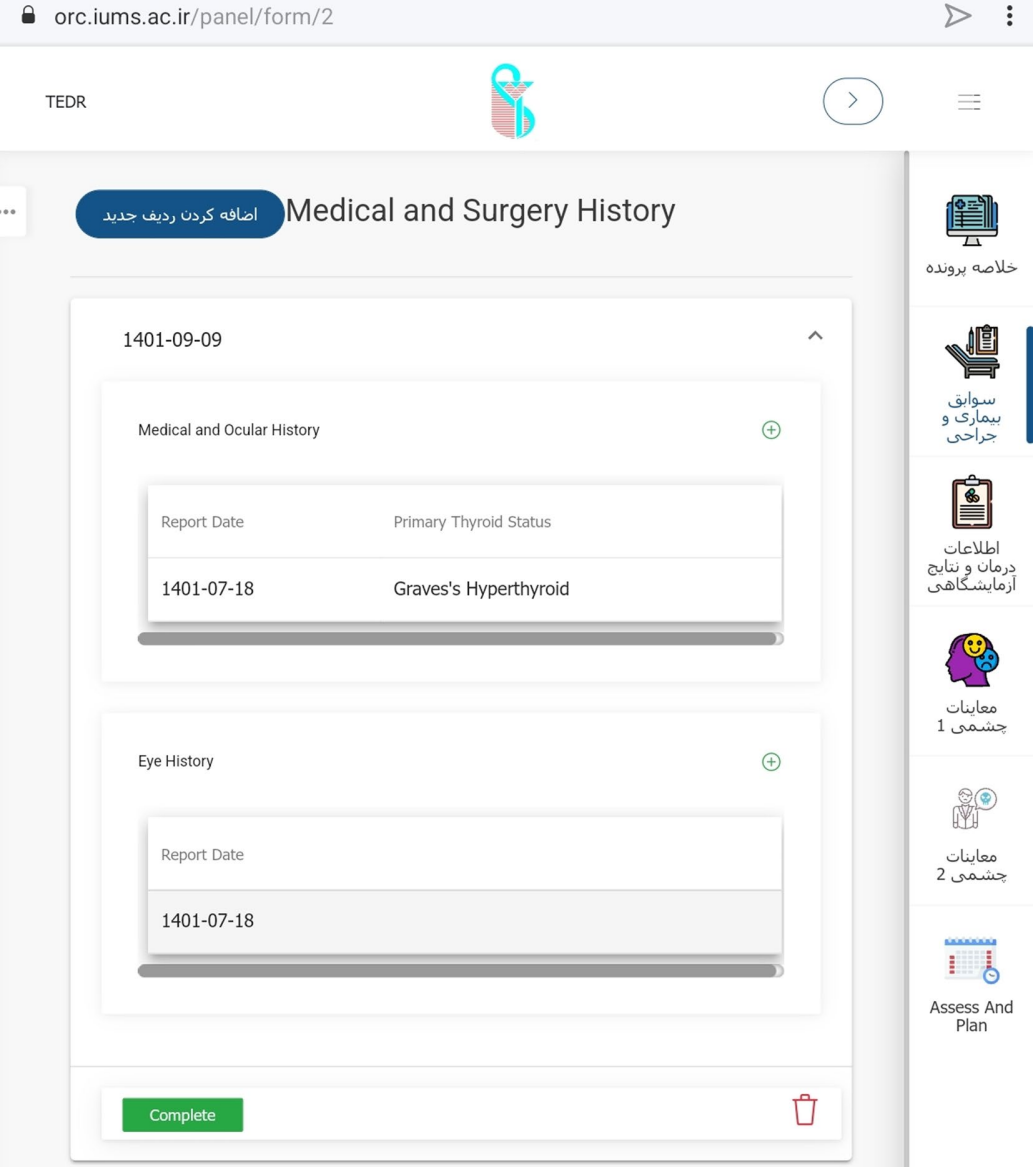

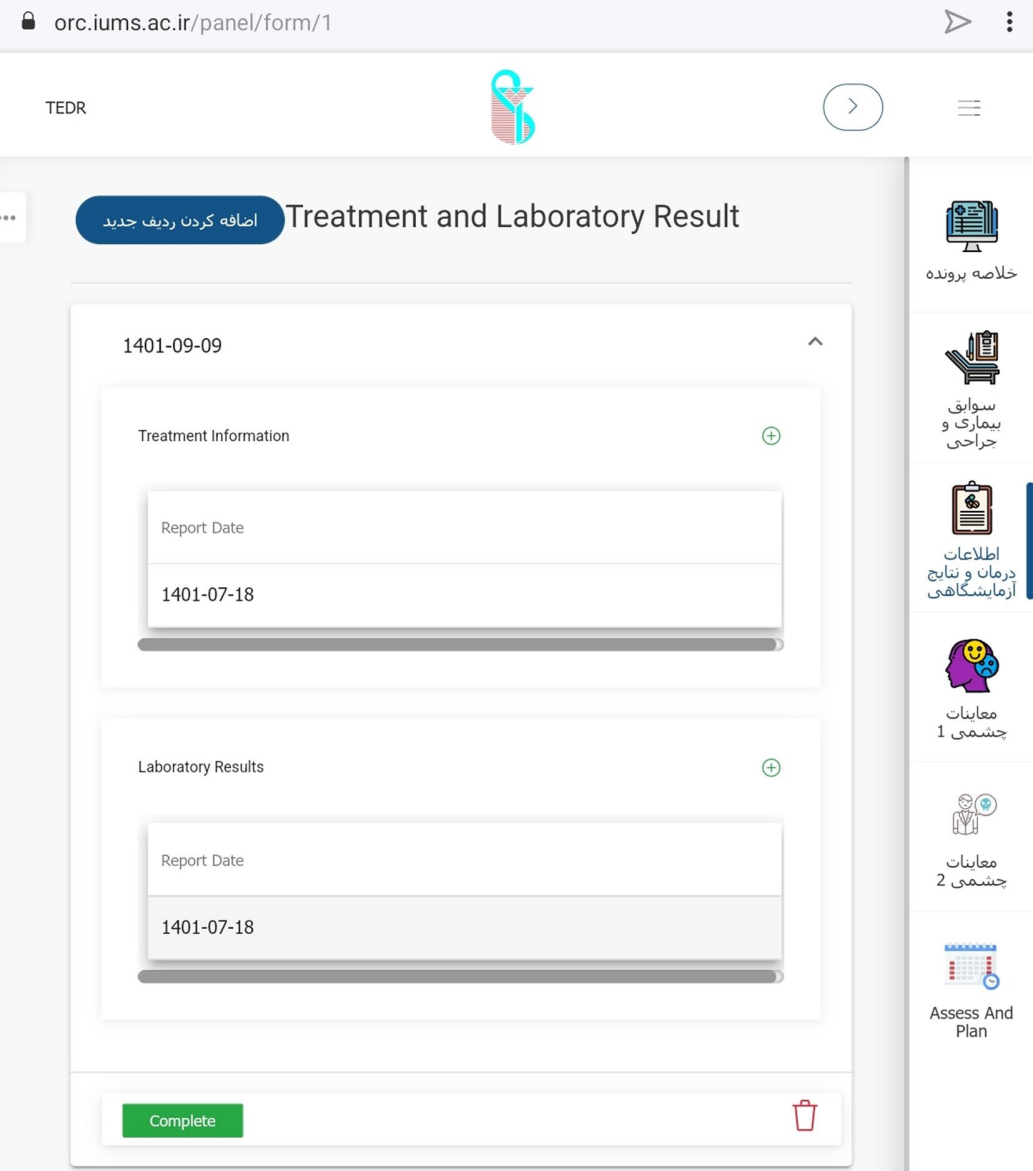

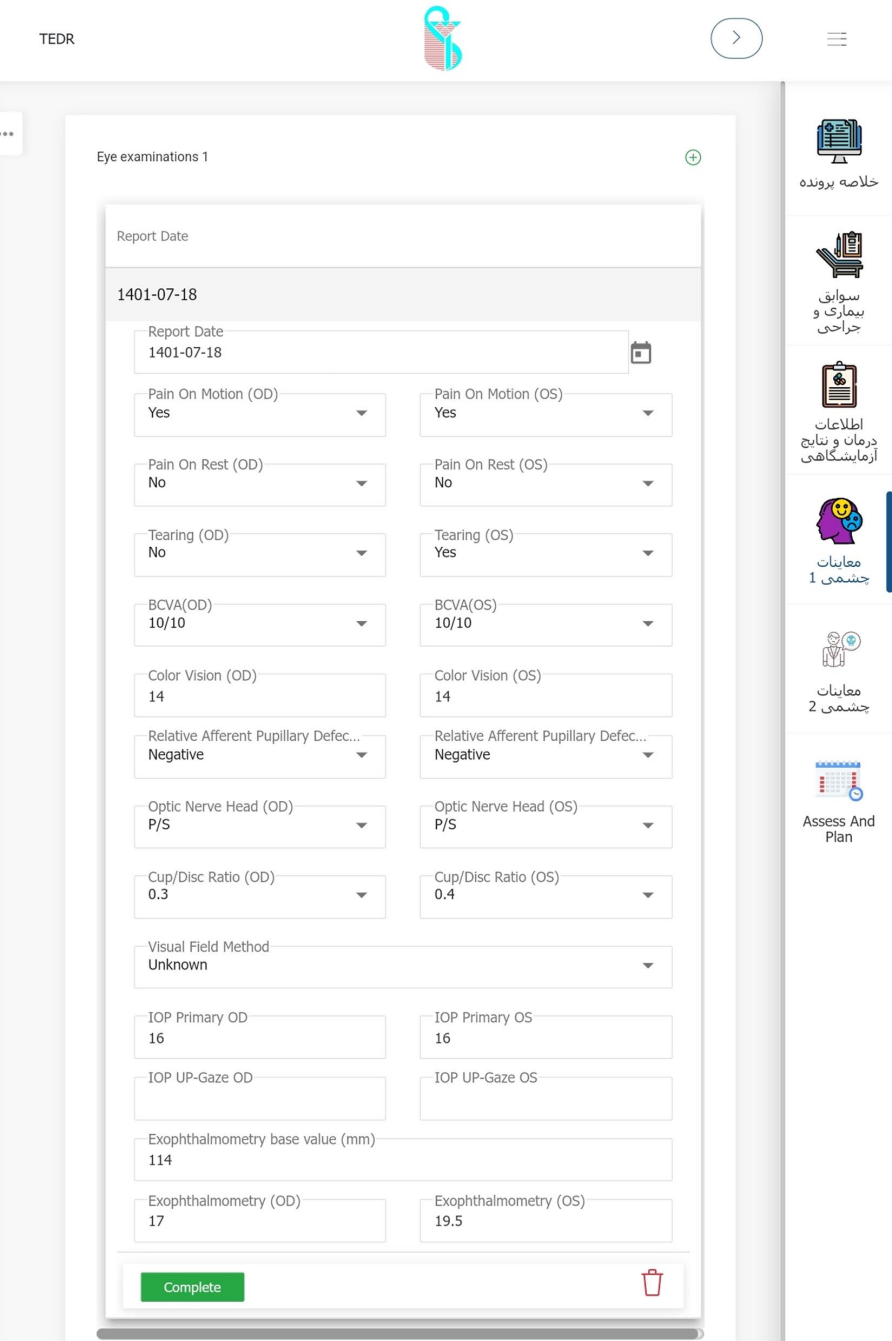

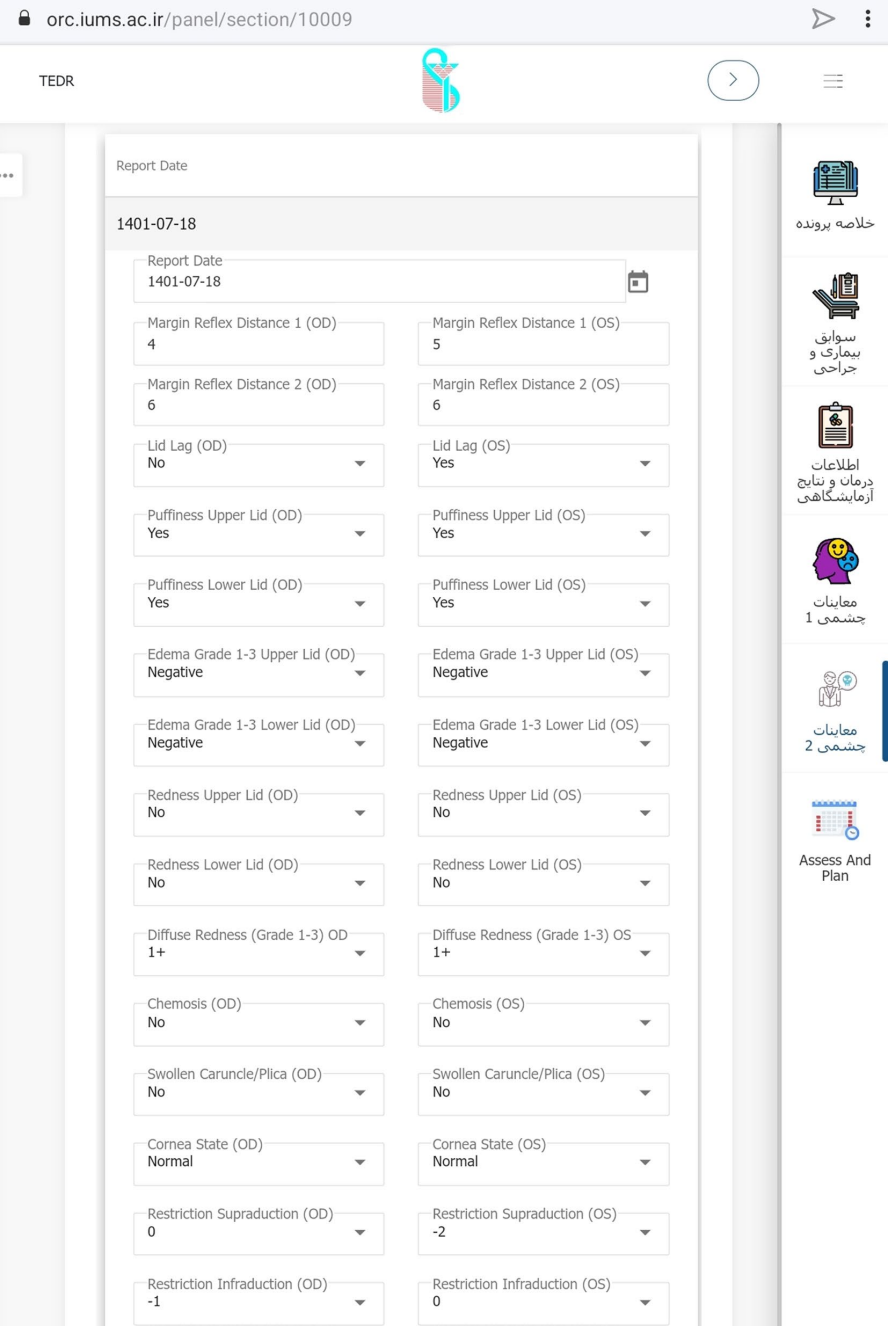

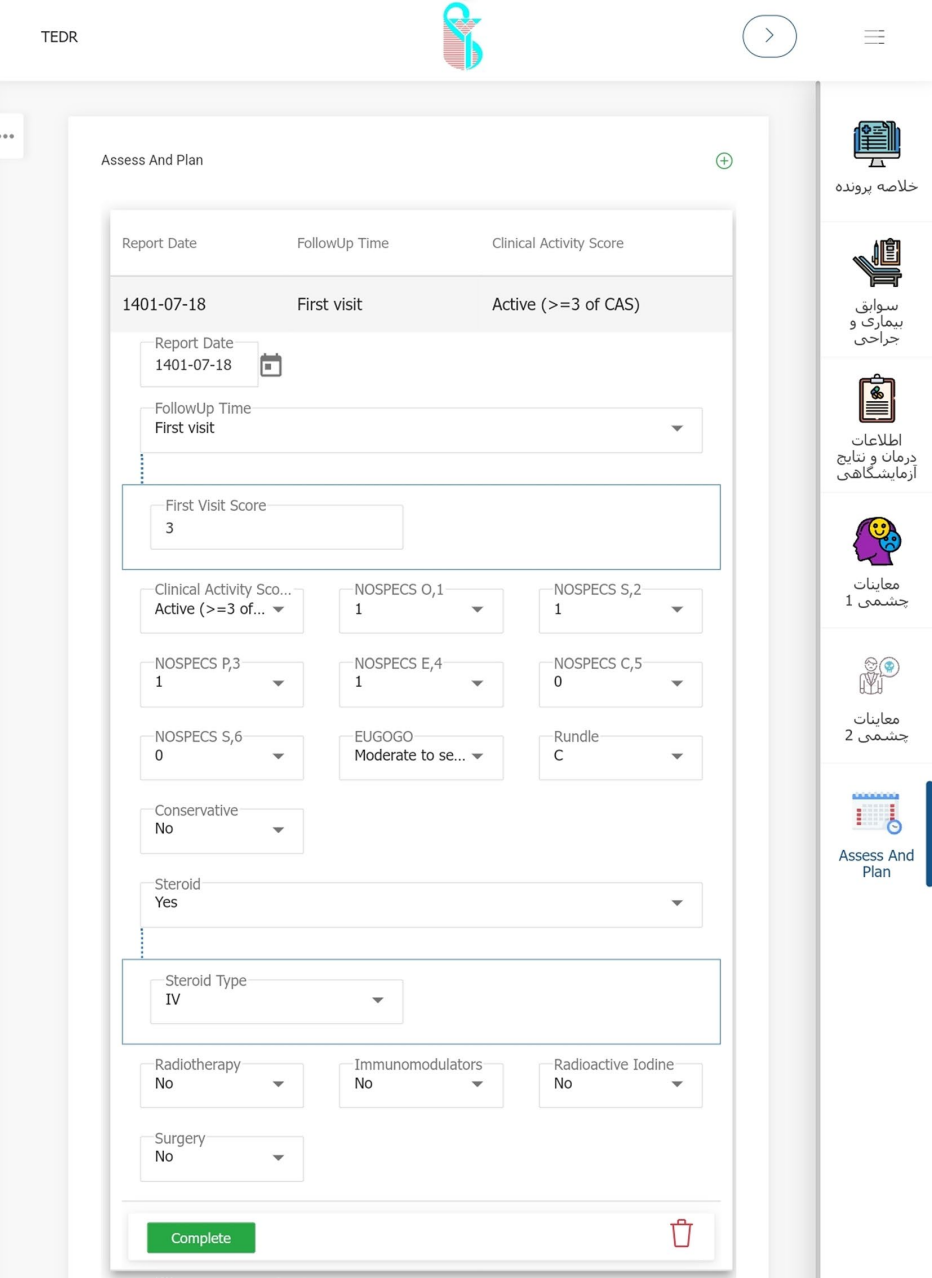
A scene shot of the software

A private company developed the software using Microsoft SQL Server 2019 and Microsoft Visual Studio 2019 using C#.Net with Net Core 3.1. The company is overseen by IUMS and required to comply with security protocols for data, hosting, and server that belong to the university. During several meetings between steering committee members and the technical staff, it received official approval from the committee. The system is currently available at https://orc.iums.ac.ir. (Fig. 4).

Access policy was also defined to maintain data security so that the user access was prioritized (based on Microsoft identity) for different members by their roles in the registry. While the principal investigator and administrative director have access to all parts of the software and data, other users’ access is limited to their roles.

#### Data collection and assurance

In every step of data collection, entry, and registration, quality control is implemented to improve data accuracy. The collected data is triple checked before and after registration; by a clinician and a trained person during the examination and an ophthalmic plastic reconstructive subspecialist after recording. All necessary clinical and para-clinical examination tools and methods and step-by-step diagnostic procedures, were standardized in the guideline approved by the steering committee.

Diverse methods have been employed for data quality checks. First, experienced clinical staff are instructed to assess and control data quality for both retrospective and prospective enrolled patients. Since data related to recruited patients before launching the project are also registered, the scanned paper-based medical records of retrospective subjects admitted to the outpatient clinic of ophthalmic plastic surgery in Rassoul Akram Hospital were evaluated and registered after quality control. If a possible error is detected, feedback are sent to authorities by the trained staff to check documents and correct them. Moreover, validation rules have also been applied in the software system to prevent recording inaccurate or incomplete data so that limited characters or compulsory data items have been defined and data consistency between related fields is checked. Additionally, all users are required to send their practical suggestions as correctional feedback to the steering committee, and if the request seems justified after analysis, the modification will be applied.

#### Statistical considerations

Conventional estimations to define sample size are not applied since this registry is not hypothesis-based. Thus, the reports will provide epidemiological features of the disease, such as prevalence, incidence, and other descriptive values, as well as the distribution of patients per area of residence.

#### Publication plan

The entered data is extracted every four months to check the frequency of missed or incorrect data and find a solution, and to be forwarded to the statistical group of the steering committee for further evaluation and analysis. Then, the latest results will be represented through diverse paper publications based on different available data. Moreover, the updated reports are sent to the disease registry committee of IUMS twice a year and the disease registry committee of MOHME yearly.

#### Ethical statement

Initially, this study received ethics approval from the Research Ethics Committee of Iran University of Medical Sciences to start the first phase of the project, and after getting approved by MOHME’s registry committee, it received the second approval number to start a collaboration with other provincial participant centers (IR.IUMS.REC.1396.32991 and IR.IUMS.REC.1400.919, respectively).

## Discussion

The national thyroid eye disease registry is a multi-center system-based patient registry that was first established in Rassoul Akram Hospital in 2019 and aimed to involve other teaching hospitals in other provinces in the country in the next phases. Collecting demographic, hereditary, and clinical data to investigate the disease's epidemiological features are our registry's main goals. A multi-center registry is applied to establish a widely applicable classification system to predict outcomes in intended patients, as well as introduce a contemporary process in clinical practice, and also estimate the effectiveness of various therapeutic methods on patients of different regions. [31] In other words, behavioral patterns of diverse populations in different geographic locations and relative outcomes are monitored. Furthermore, this provides health policymakers with an appropriate circumstance in which the variability in care is evaluated all over the country based on the number of patients and hospital types in addition to geography. [32]

Since the commencement of the patient registration in Rassoul Akram Hospital, 610 retrospective and newly diagnosed individuals have been recorded whereas 72 variables were applied to define their status. The data elements of our case report form were updated according to the latest updates on the TED form in panel discussion sessions held by the steering committee, and the following modifications were applied:

1. Adding “City of birth” and “City of location” as well as “Ethnicity” to the demographic and identification section to better understand the origin of the disease since Tehran is the capital city and patients may have moved or be referred.
2. Differentiating the type of smoking or drug usage as “Tobacco, Opioids, Cannabis” to demographic data and smoking status categories to trace them in the long-term investigation.
3. Adding “Hyperlipidemia” and “Autoimmune vasculopathy” to the general medical history section.
4. Adding “Proptosis”, “Red Eye”, “Lid Retraction”, “Foreign body sensation”, and “Dry eye” to the ocular history section as early signs and symptoms of the disease.
5. Adding “Lagophthalmos” and “orbital CT/MRI” to the visual and ocular exams section.

These changes have been adopted in both paper and software versions of TED form since the commencement of the project for new patient registration.

### Limitations and strengths

As mentioned before, the collected data is checked three times by a clinician and a technician during the examination, as well as an ophthalmic plastic subspecialist after recording. Yet, there are inevitable limitations in every step, as expected in all studies, mainly disease registries [33, 34] and tried to be detected in primary stages as much as possible and corrected manually by involved individuals or by customizing the software system. For instance, data from extracted medical records may be incomplete due to data collection limitations from retrospective paper records or insufficient data sources in hospitals. Also, due to recall bias, self-declared data relating to hereditary and general/ocular history can be potentially inaccurate. This problem could be compensated for in follow-up sessions by requesting patients to present their previous documentation. However, the lack of patients’ participation in follow-up sessions in such a circumstance will simultaneously exacerbate this situation as a restriction. Considering patients’ interests and needs as well as providing optimum knowledge about their disease and its perspective, may help patients feel more desire to participate in examinations, especially for newly diagnosed cases. Moreover, the determination of mandatory data items and the organization of the variables is another consideration. By increasing the number of data variables and defining the high volume of data elements, many items may not be completed when examining the patients and filling out the forms. So, conducting different meetings with experts to analyze and debate on finalizing the minimum mandatory items and their organization is a solution. Furthermore, since checking the quality of data, sending feedback, and correcting is time-consuming, devoting a well-trained person to supervise this flow is necessary. [33, 34] Also, in each medical center, only one user is permitted to enter data to control selection bias. Data extraction permission is given to one technician to maintain data validity. In addition, the heavy workload in the clinic may prevent practitioners from completely filling out forms and lead to missing data. A similar circumstance arises when the staffs lose their incentive for valid data entry as time passes through longitudinal studies and feel irresponsible compared to the commencement of the study. [35] Setting financial/non-financial motivations for registry individuals under their roles and performances will encourage them to enter data more precisely and accurately.

On the other hand, selecting specific academic referral hospitals compared with the country's whole population would highlight potential restrictions, including selection bias and generalizability obstacles due to low patient volume. [32] Therefore, we decided to improve the registry network in the next phases to increase the number of patients under the umbrella of our registry nationwide. Furthermore, the feasibility of administration of a new international phase (Phase 4) was also negotiated in panel discussion sessions, to be considered in the near future at the end of the national phase to follow and develop our registry purposes in a broader scope.

## Conclusion

This registry system is a powerful tool that enables us to study epidemiology and outcomes and monitor the course of thyroid eye disease as well as the appropriateness of treatments and cares delivered to patients to enhance their quality of life on a long-term basis. We included standard data elements according to the latest international updates in six classes of identification data and demographics, thyroid gland disease status, general medical history, visual and ocular examination data, and scoring criteria to reveal TED status.

Using a customized web-based software system that provides our practitioners with valid and reliable data collection supplies an environment to have a superior investigation of outcomes and a better analysis of challenges in the next phases. Furthermore, it allows the data to be exchangeable, especially when the scope of the study is broadened in collaboration with further centers in national and international phases in the near future, and comparable with other TED registries. This also gives a profound insight to other decision-makers as involved multidisciplinary stakeholders to manage to fund for organization coordination and their linkage, and consequently run workshops to instruct all purposes and phases to healthcare professionals and then plan programs for enhancing patients’ quality of life for the future.

## Data Availability

Data sharing does not apply to this article as we have not analyzed any data during the current study.

## Abbreviations

TED: Thyroid eye disease
IUMS: Iran University of medical sciences
MOHME: Ministry of health and medical education
